# Integrative metabolome and transcriptome analyses reveal the molecular mechanism underlying variation in floral scent during flower development of *Chrysanthemum indicum* var. *aromaticum*

**DOI:** 10.3389/fpls.2022.919151

**Published:** 2022-09-15

**Authors:** Lu Zhu, Jiahao Liao, Yifei Liu, Chunmiao Zhou, Xu Wang, Zhigang Hu, Bisheng Huang, Jingjing Zhang

**Affiliations:** ^1^College of Pharmacy, Hubei University of Chinese Medicine, Wuhan, Hubei, China; ^2^South China Botanical Garden, Chinese Academy of Sciences, Guangzhou, China

**Keywords:** *Chrysanthemum indicum* var. *aromaticum*, volatile metabolites, metabolome, transcriptome, floral scent

## Abstract

*Chrysanthemum indicum* var. *aromaticum* (CIA) is an endemic plant that occurs only in the high mountain areas of the Shennongjia Forest District in China. The whole plant, in particular the flowers of CIA, have intense fragrance, making it a novel resource plant for agricultural, medicinal, and industrial applications. However, the volatile metabolite emissions in relation to CIA flower development and the molecular mechanisms underlying the generation of floral scent remain poorly understood. Here, integrative metabolome and transcriptome analyses were performed to investigate floral scent-related volatile compounds and genes in CIA flowers at three different developmental stages. A total of 370 volatile metabolites, mainly terpenoids and esters, were identified, of which 89 key differential metabolites exhibited variable emitting profiles during flower development. Transcriptome analysis further identified 8,945 differentially expressed genes (DEGs) between these samples derived from different flower developmental stages and KEGG enrichment analyses showed that 45, 93, and 101 candidate DEGs associated with the biosynthesis of phenylpropanoids, esters, and terpenes, respectively. Interestingly, significant DEGs involved into the volatile terpenes are only present in the MEP and its downstream pathways, including those genes encoding ISPE, ISPG, FPPS, GPPS, GERD, ND and TPS14 enzymes. Further analysis showed that 20 transcription factors from MYB, bHLH, AP2/EFR, and WRKY families were potentially key regulators affecting the expressions of floral scent-related genes during the CIA flower development. These findings provide insights into the molecular basis of plant floral scent metabolite biosynthesis and serve as an important data resources for molecular breeding and utilization of CIA plants in the future.

## Introduction

Floral scent is a critical phenotypic trait for flowering plants in terms of their reproductive and ecological success (Gang, 2005). In ornamental horticulture, floral fragrance has an important effect on customer choice and has great aesthetic and economic values (Oliva et al., 2015). Floral scent derives from a series of volatile secondary metabolites with low molecular weights and boiling points, including terpenoids, benzene/phenylpropanoids, fatty acid derivatives, and sulfur and nitrogen compounds (Kutty and Mitra, 2019; Maiti and Mitra, 2019). To date, about 2000 floral volatile compounds have been identified in plants, and many of them have been widely used in medicinal and cosmetic products, including perfumes and food flavorings (Knudsen et al., 2006).

The biosynthetic pathways of some floral volatile compounds have been studied in plants (Pichersky and Dudareva, 2007; Maeda and Dudareva, 2012; Kumari et al., 2013; Zhu et al., 2019). Terpenes synthesis occurs through two independent pathways, including the cytoplasmic mevalonate (MVA) pathway and the plastid 2-C-methyl-D-erythritol 4-phosphate (MEP) pathway, and the formation of various terpenes is directly catalyzed by diverse terpene synthases (TPSs) and further modified by tailoring enzymes to diversify scaffolds (Dudareva et al., 2013; Karunanithi and Zer Be, 2019). Benzene/phenylpropanoid compounds are synthesized by the cinnamic acid pathway (Dudareva and Pichersky, 2000). The formation of fatty acid derivatives generally uses membrane lipids as precursors, and hydroperoxides are further produced through the activity of lipoxygenase (LOX) on linolenic and linoleic acids. Finally, the formation of 6-carbon and 9-carbon aldehydes is catalyzed by hydroperoxidase lyase (HPL; Feussner and Wasternack, 2002), and the corresponding alcohols are formed through the catalysis of ethyl alcohol dehydrogenase (ADH; Feussner and Wasternack, 2002; Schwab et al., 2008). Additionally, transcription factors (TFs), such as those from ethylene resposive factor (*ERF*), basic helix–loop–helix (*bHLH*), and v-myb avian myeloblastosis viral oncogene homolog (*MYB*) families, are also crucial to regulate the biosynthesis of floral volatiles (Jian et al., 2019; Ramya et al., 2019).

The complex biochemical pathways of floral aroma synthesis are dominated by a variety of internal and external stimuli, such as the circadian clock, the environment, and different stages of flower development, leading to controlled emissions of volatile compounds (Van et al., 2009; Abbas et al., 2017). To maximize pollination opportunities and minimize the chances of injury, flowers tend to start producing fragrance when they are ready to receive pollen and when potential pollinators are most active. In most plants, floral fragrance gradually diminishes and disappears after the flowers have been pollinated (Yakir et al., 2010). Environmental factors including illumination and temperature can also affect the presence of floral scent. Plant 5-phosphate deoxyxylulose reductive allosterase (DXR) only participates in the phosphodeoxyxylulose (DXP) pathway in the light, and illumination can therefore affect the activity of this pathway and the accumulation of monoterpenes (Lichtenthaler et al., 1997). The release of volatiles in *Trifolium repens* flowers was significantly lower at 10°C than at 15 and 20°C, suggesting that an increase in temperature could accelerate volatile release (Olsen, 1994). Among different flower developmental stages, scent emission is generally higher in half-open and fully open flowers (Mohd-Hairul et al., 2010). For example, in *Polianthes tuberosa*, low levels of benzenoids and terpenes were detected in green flower buds, but their presence increased at flower anthesis (Fan et al., 2018a).

*Chrysanthemum indicum* var. *aromaticum* (CIA) is a plant that naturally occurs in the high-altitude (>2000 m above sea level) mountains of the Shennongjia Forest region in Hubei Province, China (Liu et al., 1983; Liu and Zhang, 1983). It is a variety of *C. indicum* (CI) and morphologically closely related to CI, but there are clear differences between both taxa. Compared with CI, CIA is relatively small in plant size, given its long-term adaptation to high-altitude environments, and the whole CIA plant, especially the flowers, releases an intense fragrance. The aroma substances in CIA have been widely studied by gas chromatography–mass spectrometry (GC–MS), and terpenes are the main volatile components (Lin et al., 2016; Fan et al., 2018b). The essential oil and concrete extracted from CIA have already been used as additives in perfumes, cosmetics, and health products (Zhu et al., 2012). Moreover, CIA is an ideal germplasm material for breeding aromatic cultivars of both ornamental and medicinal chrysanthemums (Wang et al., 2021). Despite progress in investigating the volatile components, little is known about the transcriptional regulation and emission in CIA floral scent at different flower developmental stages.

In recent years, the integration of metabolome and transcriptome analyses has become a powerful way to characterize the specialized metabolism network of volatile organic compounds in vegetables and flowers (Zhu et al., 2019; Kutty et al., 2021). In this study, to determine the emission patterns of scent volatiles in relation to flower development, we analyzed the volatile compound profiles of CIA flowers from three developmental stages by headspace solid-phase microextraction gas chromatography–mass spectrometry (HS-SPME-GC–MS), and investigated the expression patterns of floral scent-related genes by RNA-seq. We revealed the dynamic changes of aroma substances during CIA flower development and characterized the expression pattern of genes associated with key differential metabolite. The results improve the understanding of the molecular mechanisms of CIA aroma compounds and the breeding and product development of CIA.

## Materials and methods

### Plant materials

All CIA plants used in the present study were planted in the same place of the Shennongjia Forestry district of Hubei Province, China (110°23′57″E, 31°28′7″N). Based on the bloom stage of CIA flowers, samples of flower buds (calyx dehiscent, outer whorl petals involute; FB), initial flowers (only the outer 3–4 layers of petals unfolded, the remaining petals still wrapped tightly; IF) and blooming flowers (ligulate flowers all in full bloom; BF) were collected in October 2020 (Supplementary Figure 1). For each flower development stage investigated, three replicates were immediately frozen in liquid nitrogen and stored at −80°C for transcriptome profiling by RNA-seq, and six replicates were stored at −20°C for analysis of volatile compounds by HS-SPME-GC–MS.

### HS-SPME-GC–MS

We used the SPME cycle of the PAL rail system; the incubation temperature was 60°C, the preheating time 15 min, the incubation time 30 min, and the desorption time 4 min. GC–MS analysis was performed using an Agilent 7,890 gas chromatograph system coupled with a 5977B mass spectrometer. The system utilized a DB-Wax column injected in splitless mode. Helium was used as the carrier gas, the front inlet purge flow was 3 ml/min, and the gas flow rate through the column was 1 ml/min. The initial temperature was kept at 40°C for 4 min, then raised to 245°C at a rate of 5°C/min and held for 5 min. The injection, transfer line, ion source, and quad temperatures were 250, 250, 230, and 150°C, respectively. The energy was −70 eV in electron impact mode. The mass spectrometry data were acquired in scan mode with an m/z range of 20–400 and a solvent delay of 0 min. NIST08 library was used to identify volatiles based on comparing the mass spectrometry and retention indices of the compounds detected with existing data available in it.

### Metabolome data analysis

The filtered data were submitted to Simca-P software (version 13.0, Umetrics AB, Umea, Sweden) for unsupervised principal component analysis (PCA) and orthogonal partial least squares-discriminant analysis (OPLS-DA). Hierarchical clustering analysis of the metabolites between the samples was carried out with TBtools software.^1^ To identify differentially accumulated metabolites (DAMs), |log2FoldChange| ≥ 1 and variable importance in projection (VIP) ≥ 1 were used as the screening criteria.

### RNA extraction, cDNA library preparation, and RNA sequencing

Total RNA was isolated from the FB, IF, and BF samples with an RNA extraction kit (Cat. No. RP3202, BioTeke Corporation, Beijing, China). RNA degradation and contamination were monitored on 1% agarose gels. RNA purity was checked using a NanoPhotometer spectrophotometer (IMPLEN, CA, United States). RNA integrity was assessed using the RNA Nano 6,000 Assay Kit with a Bioanalyzer 2,100 system (Agilent Technologies, CA, United States). A total amount of 1 μg RNA per sample was used as input material for RNA library preparation. Sequencing libraries were generated using the NEBNext Ultra RNA Library Prep Kit for Illumina (NEB, United States) following the manufacturer’s recommendations, and index codes were added to associate sequences with specific samples. To preferentially select cDNA fragments 250–300 bp in length, the library fragments were purified with the Ampure XP system (Beckman Coulter, Beverly, United States). Then 3 μl USER Enzyme (NEB, United States) was used with size-selected, adaptor-ligated cDNA at 37°C for 15 min, followed by 5 min at 95°C before PCR. PCR was performed with Phusion High-Fidelity DNA polymerase, Universal PCR primers, and Index (X) Primer. PCR products were purified (Ampure XP system), and library quality was assessed on the Agilent Bioanalyzer 2,100 system. Finally, the libraries were sequenced on the Illumina NovaSeq platform to generate 150-bp paired-end reads.

### Transcriptome analysis

Clean reads were obtained by removing reads that contained adapters, reads that contained poly-N, and low-quality reads (>50% bases with Qphred ≤20). All downstream analyses were performed using the high-quality, clean data. Clean reads were assembled using Trinity^2^ (Grabherr et al., 2011). The longest cluster sequence was obtained by Corset hierarchical clustering and used in subsequent analyses^3^ (Davidson and Oshlack, 2014). Statistics were calculated for the lengths of the transcripts and cluster sequences. The prediction of coding sequences was carried out by performing alignment according to the priority order of NR protein library and Swissprot protein library. If the alignment result is available, the ORF coding frame information of the transcript was extracted and the coding region sequence was translated into amino acid sequence according to the standard codon list (in the order of 5′→3′). The Estscan (3.0.3) software was used to predict their ORF of unalignment or unpredicted sequences. To obtain comprehensive gene function information, gene annotations were obtained from seven databases: Nr (NCBI non-redundant protein sequences), Nt (NCBI non-redundant nucleotide sequences), Pfam (Protein family), KOG/COG (Clusters of Orthologous Groups of proteins), Swiss-prot (a manually annotated and reviewed protein sequence database), KEGG (Kyoto Encyclopedia of Genes and Genomes), and GO (Gene Ontology). Fragments per kilobase of exon model per million mapped fragments (FPKM) was calculated to estimate the level of gene expression in the sample (Trapnell et al., 2010). Differential expression analysis between samples was performed using the DESeq2 R package (1.16.1; Love et al., 2014), and DEGs were identified based on the threshold of |log2 foldchange| ≥ 1 and FDR (false discovery rate) ≤ 0.05. DEGs were subjected to GO and KEGG enrichment analysis using the clusterProfiler R package (Minoru et al., 2008; Young et al., 2010). Venn diagrams were drew online^4^ and R package were used to plot the expression heatmaps (Yu et al., 2012). Pearson’s correlation between the levels of TFs expression and the content of differential metabolites and was analyzed by using R (version 4.0.3). The correlation network was plotted by Cytoscape (v3.8.2).

### Gene expression validation by qRT-PCR

The frst-strand cDNA was synthesized from the total RNA of the same samples used in RNA-seq. The TranScript One-Step gDNA Removal and cDNA Synthesis SupperMix (TransGen, China) were used. The qRT-PCR was performed on a LightCycler96® Instrument (Roche Diagnostics GmbH) with 20 μl reaction consisited of 10 μl of 2X Universal SYBR Green Fast qPCR Mix(SYBR Green I; ABclonal, China), 0.4 μl of 10 μM Primer F, 0.4 μl of 10 μM Primer R, 1 μl of cDNA template and 8.2 μl ddH_2_O. The amplification program was as follows: pre-denaturation at 95°C for 3 min, followed by 42 cycles of denaturation at 95°C for 5 s, renaturation at 60°C for 30 s, and extension at 72°C for 10 s. The chrysanthemum *CmEF1α* (GenBank ID, KF305681.1) was used as a reference gene, and the relative expression levels were calculated according to 2^−ΔΔC*t*^ method (Lalitha, 2000). Three biological replicates were used and the primers used for these validations were shown in Supplementary Table 1. The statistical value of *p* was generated by the Student’s *t*-test. The statistical significance was defined as *p* < 0.05.

## Results

### Variation in volatile metabolites during CIA flower developmental stages

**Figure 1:**
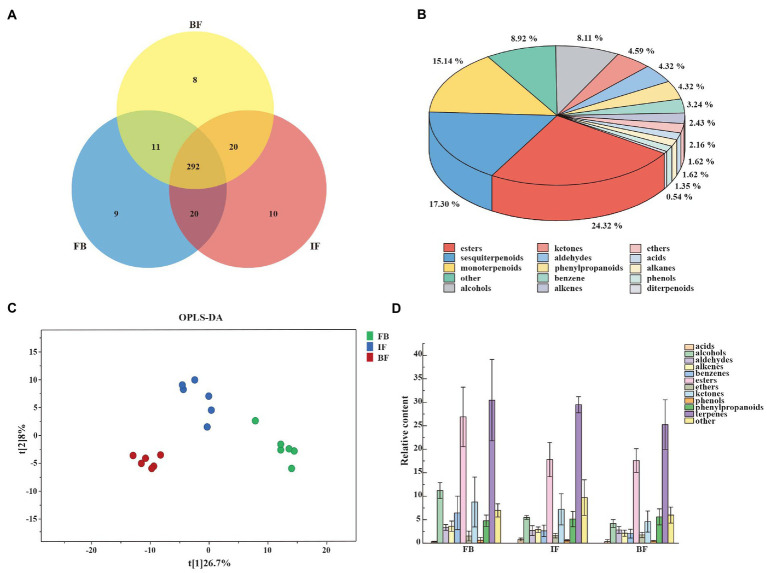
Characterization of volatile compounds identified in *Chrysanthemum indicum* var. *aromaticum* (CIA) flowers. **(A)** Venn diagram of volatile compounds in CIA flowers. **(B)** General classification of volatile substances. **(C)** OPLS-DA score plots showing clusters of CIA flower samples (*n* = 3). **(D)** Trends in the release of volatile metabolites during flower opening at the flower bud (FB), initial flower (IF), and blooming flower (BF) stages.

CIA flower samples from three developmental stages of FB, IF, and BF were collected for investigating volatile compound profiling using HS-SPME-GC–MS. In total, 370 volatile metabolites were identified, including 292 compounds commonly presented across all sampled stages, and 9, 10, and 8 compounds uniquely presented in FB, IF, and BF samples, respectively (Figure 1A; Supplementary Table 2). These metabolites can be categorized into 15 classes, with the main classes of terpenoids (34.87%), followed by esters (24.32%) and others (8.92%; Figure 1B; Supplementary Table 3). For the volatile terpenoids identified, both sesquiterpenoids (17.3%) and monoterpenoids (15.14%) are the majority.

**Figure 2:**
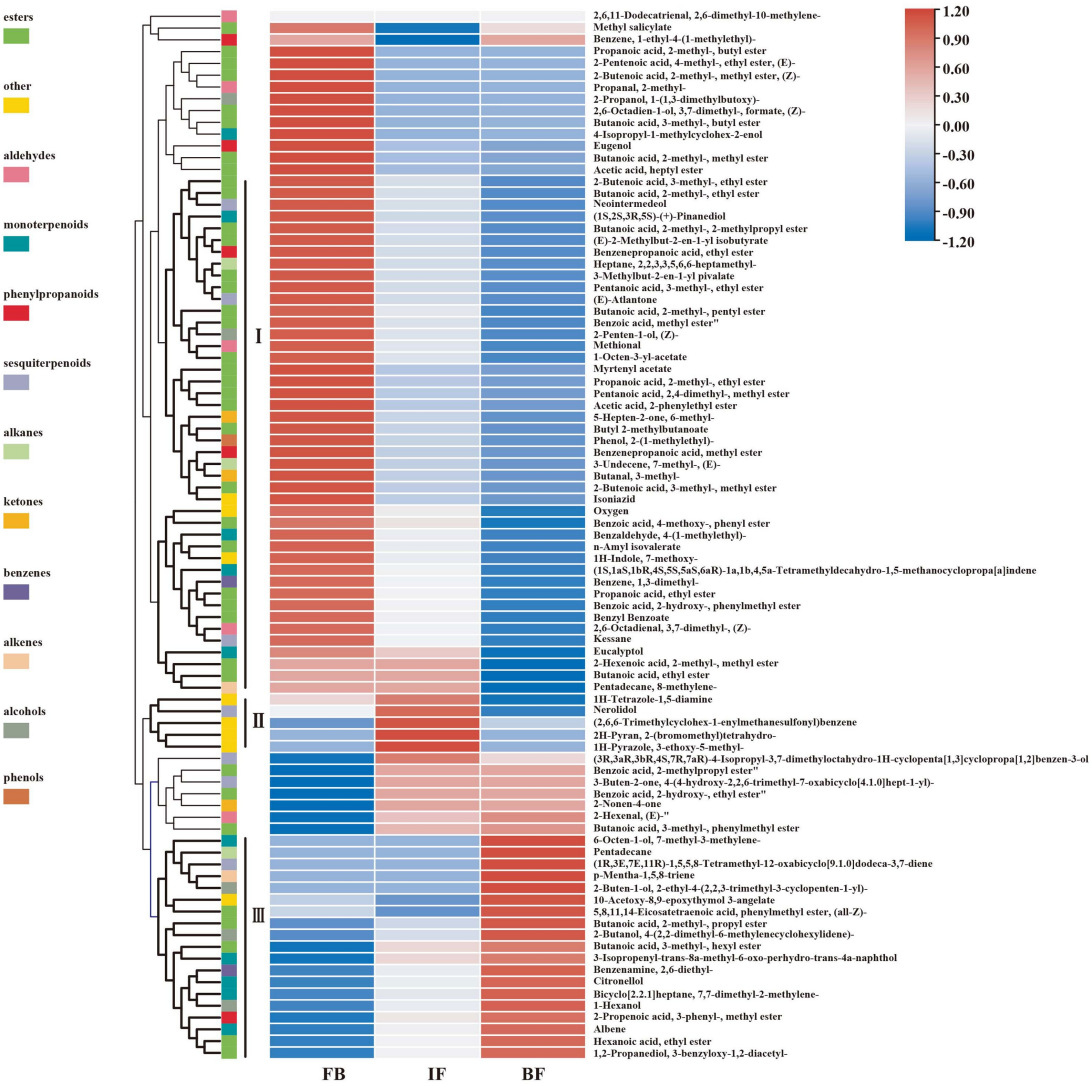
Hierarchical clustering heat map of 89 differential volatile metabolites at three floral developmental stages of *Chrysanthemum indicum* var. *aromaticum*.

Differential metabolites between three different flower development stages were analyzed by using orthogonal partial least squares-discriminant analysis (OPLS-DA). When a volatile metabolite has a variable importance in projection (VIP) value ≥1, it is considered as a key differential metabolite between samples. A total of 89 key differential metabolites were identified, including 26, 78, and 17 presented between FB and IF, FB and BF, and IF and BF samples, respectively (Supplementary Tables 4–7). These differential metabolites clustered into three groups on the OPLS-DA plate, suggesting the difference between the volatile metabolite profiles of stage samples (Figure 1C). Comparative analysis showed that the relative contents of some floral scent compounds decreased as the flowers gradually opened, whereas others showed the opposite pattern (Figure 1D). For example, we found 44 volatile compounds which presented relatively high contents in the FB samples, but were less present in the IF samples, and some were even not detected in the BF samples (Figure 2 I). Similarly, five volatile compounds present mainly in the IF samples, and 19 compounds with increased emission in the BF stage (Figure 2 II–III; Supplementary Tables 4–7).

We further filtered 39 major differential compounds with their relative contents >1% in at least one of the three investigated stages (Supplementary Table 8). During the flower development stages, the contents of 22 major compounds, including 6 esters, 4 monoterpenoids, 2 alcohols, 1 ketone, 1 aldehyde, 1 phenylpropanoid, 2 benzenes, and 1 alkene, gradually decreased, while only those of linalool and 3-decyn-2-ol gradually increased. Interestingly, the emission of butanoic acid, 2-methyl-, 2-phenylethyl ester, methyl salicylate, 1-(1-cyanocyclopentyl)pyrrolidine, and benzene, 1-methyl-3-(1-methylethyl)- decreased from FB to IF stages, but slowly increased at the BF stage. On the contrary, the contents of 11 compounds such as (1R,2R,5S)-5-methyl-2-(prop-1-en-2-yl)cyclohexanol, butanoic acid, 2-methyl-, phenylmethyl ester, 2-cyclohexen-1-ol, methyl alcohol, tetramethylbicyclo[7.2.0]undeca-2,6-diene, junenol, cyclobutanone, and 2-Propenoic acid, 3-phenyl-, methyl ester, (E)-, increased from the FB to the IF stages, but decreased at the BF stage. Collectively, these results suggested that the main floral volatile metabolites changed during CIA flower development, including some stage-specific compounds.

### Transcriptome assembly and functional annotation

**Figure 3:**
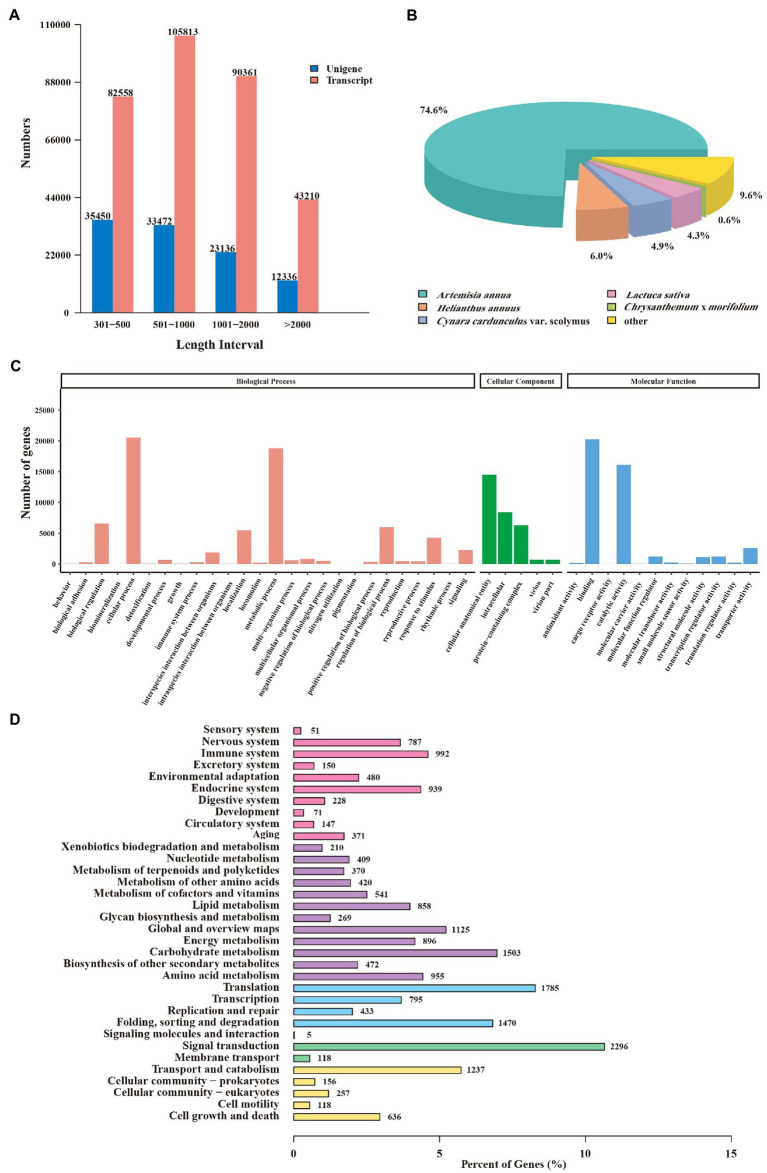
Annotation and functional analysis of unigenes from *Chrysanthemum indicum* var. *aromaticum* flowers. **(A)** The length distribution of transcripts and unigenes. **(B)** Species annotations of unigene homologs. **(C)** Gene ontology terms of unigenes. **(D)** Unigenes annotated in the KEGG pathways.

RNA sequencing (RNA-seq) was performed to profile the global transcriptome of CIA flower samples from three developmental stages. A total of 19.55 Gb, 20.07 Gb, and 19.79 Gb clean Illumina reads were retained from the FB, IF, and BF samples, respectively (Supplementary Table 9). The percentages of bases with quality >20 (Q20) and >30 (Q30) were over 97 and 91.9%, respectively. The percentages of the GC contents of the clean reads ranged from 41.65 to 41.87%. These results indicated the high quality of the transcriptome data which was suitable for the next analysis. After *de novo* assembly, a total of 321,942 transcripts and 104,394 unigenes were obtained. Among them, 33.96% of the unigenes were 300–500 bp in length, 32.87% of the transcripts were 500–1,000 bp in length, and 133,571 transcripts (41.49%) and 35,472 unigenes (33.98%) were > 1 kb in length (Figure 3A).

A total of 55,737 (53.39%) unigenes were functionally annotated, including 6,028 (5.77%) were annotated in nine RNA-seq databases, and 65,106 (62.36%) were annotated in at least one database (Supplementary Table 10). According to the matched species distribution in different databases, the top five species were *Artemisia annua* (41,587, 74.6%), *Helianthus annuus* (3,341, 6.0%), *Cynara cardunculus* var. *Scolymu* (2,725, 4.9%), *Lactuca sativa* (2,388, 4.3%), and *Chrysanthemum* × *morifolium* (327, 0.6%), suggestting their close relationships with CIA (Figure 3B). Gene Ontology (GO) analysis of these unigenes further revealed 1,321 GO terms which belong to different “biological process,” “cellular component,” or “molecular function” categories (Figure 3C). “Cellular process” was the most abundant term in the “biological process” category, “cellular anatomical entity” was associated with the highest number of unigenes in the “cellular component” category, and “binding” was the top-ranked term in the “molecular function” category (Figure 3C; Supplementary Table 11).

Furthermore, 18,347 unigenes were divided into five branches and 34 metabolic maps according to the KEGG database. The “metabolism” branch had 10,797 unigenes, followed by the “organismal systems” and “genetic information processing” branches (6,269 and 5,206, respectively). Within the “metabolisms” branch, “carbohydrate metabolism” (1,503, 13.92%) contained the highest number of unigenes (Figure 3D; Supplementary Table 12). These unigenes were related to several secondary metabolite biosynthesis pathways, such as phenylpropanoid biosynthesis (258 unigenes), terpenoid backbone biosynthesis (125 unigenes), sesquiterpenoid and triterpenoid biosynthesis (66 unigenes), and isoquinoline alkaloid biosynthesis (60 unigenes; Supplementary Table 12).

### Gene expression and identification of DEGs

**Figure 4:**
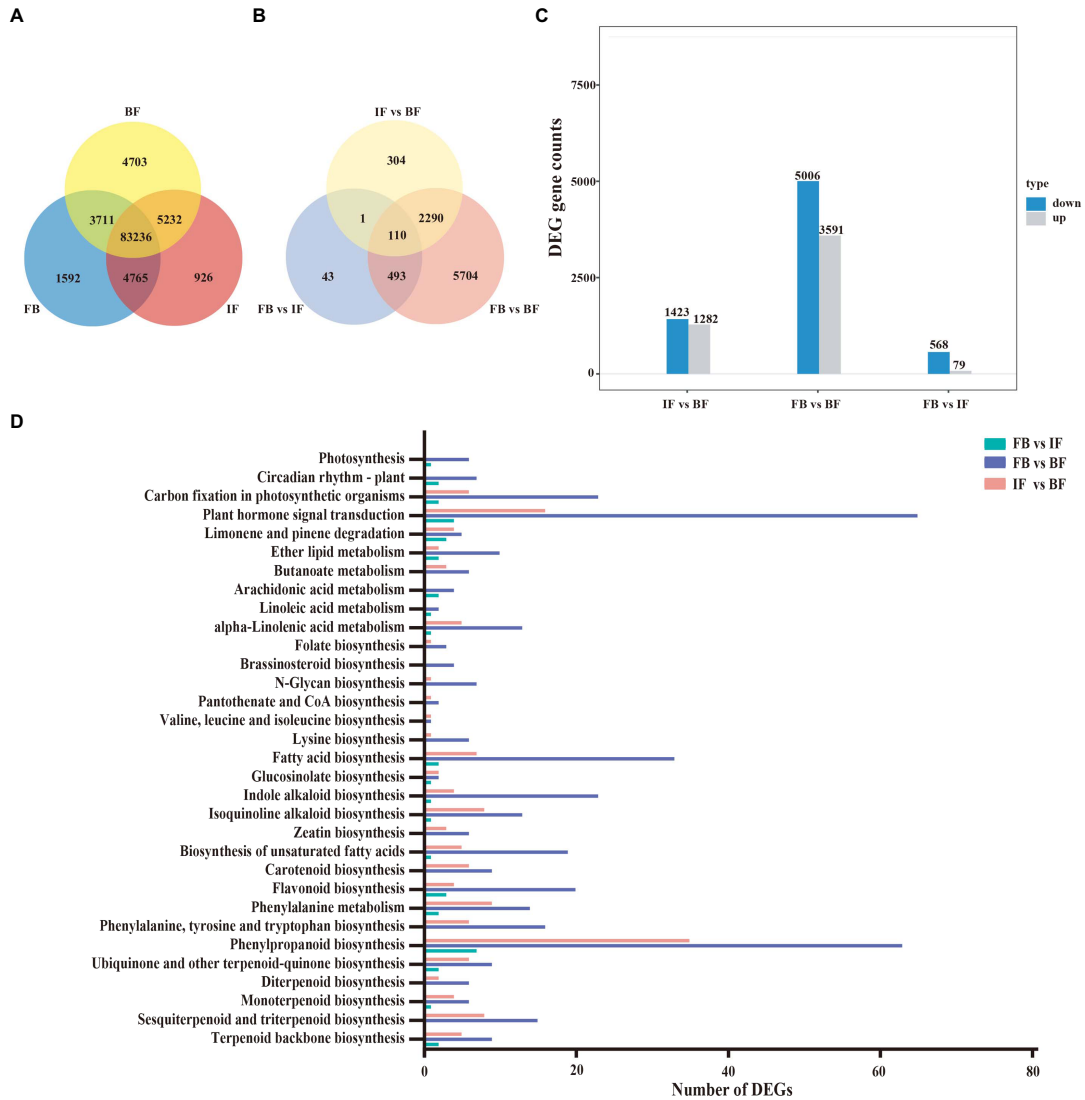
Gene expression and functional classification of DEGs in *Chrysanthemum *indicum** var. *aromaticum* flowers. **(A)** Venn diagram of genes expressed at three different flower developmental stages. **(B)** Venn diagram of differentially expressed genes from three comparisons between different floral development stages. **(C)** Histogram of up- and downregulated genes with the latter as the reference in three comparisons. **(D)** KEGG enrichment analysis of DEGs from three comparisons.

A total of 104,165 expressed genes (FPKM>0.3) were surveyed, including 93,304 genes expressed in the FB samples, 94,159 in the IF samples, and 96,882 in the BF samples. Amongst, 83,236 genes (79.9%) were expressed commonly among the flower samples from three development stages, and 1,592, 926, and 4,703 genes were specifically expressed at the FB, IF, and BF stages, respectively (Figure 4A). Pairwise comparative analysis between these stage samples revealed 8,945 significantly differentially expressed genes (DEGs), including 647 DEGs presented between the FB and IF samples, 8,597 DEGs between the FB and BF samples and 2,705 DEGs between the IF and BF samples, respectively (Figure 4B). Comparatively, more DEGs were upregulated in the three comparisons during CIA flower development from FB to BF stages (Figure 4C; Supplementary Table 13). Moreover, the relative expression profiles of DEGs appear to be diverse, with FB and IF samples clustered preferentially (Supplementary Figure 2).

KEGG enrichment analyses were performed to functionally classify these DEGs (Figure 4D; Supplementary Table 14). Seven biosynthesis pathways related to floral fragrance were identified, including “terpenoid backbone biosynthesis” (TBB: ko00900), “sesquiterpenoid and triterpenoid biosynthesis” (STB: ko00909), “monoterpenoid biosynthesis” (MB: ko00902), “phenylpropanoid biosynthesis” (PB: ko00940), “flavonoid biosynthesis” (FB: ko00941), “fatty acid biosynthesis” (FAB: ko00061), and “biosynthesis of unsaturated fatty acids” (BUFC: ko01040). Clearly, the DEGs in the three stage comparisons were distinct (Figure 4D; Supplementary Figures 3–5; Supplementary Table 14). For example, DEGs in relation to the TBB (9), MB (6), STB (15), PB (63), FB (20), FAB (33), and BUFC (19) pathways were more widely presented in the FB *vs*. BF comparison than those in the IF *vs*. BF comparison (Supplementary Figures 3, 4; Supplementary Table 14), while less related DEGs were presented in the FB *vs*. IF comparison (Supplementary Figure 5; Supplementary Table 14). Interestingly, the numbers of these floral fragrance-related DEGs are consistent with those of differential floral scent volatiles identified between different CIA flowers developmental stages, suggesting that these DEGs are potentially key genes responsible for the production of differential volatile metabolites.

### Genes involved in volatile terpenes biosynthesis

Terpenes are the main components of CIA flower volatiles, and the biosynthesis of their upstream precursors occurs through the terpenoid backbone pathway (ko00900), including the MVA and MEP pathways. No significant DEGs were detected in the MVA pathway. In the MEP pathway, one 4-diphosphocytidyl-2-C-methyl-D-erythritol kinase gene (ISPE, Cluster-15110.69196) and two 4-hydroxy-3-methylbut-2-en-1-yl diphosphate reductase genes (ISPH, Cluster-15110.25969 and Cluster-15110.41634) were identified, and their expression levels increased as the CIA flowers gradually bloomed. In the downstream biosynthetic pathway of terpenes, 17 and nine DEGs were functionally annotated and associated with sesquiterpene and monoterpene biosynthesis, respectively.

The transcript abundance of one farnesyl diphosphate synthase gene (FPPS, Cluster-15110.30808), and five (−)-germacrene D synthase genes (GERD, Cluster-15110.10761, Cluster-15110.10884, Cluster-15110.17454, Cluster-3197.0, and Cluster-8673.0) showed high levels in flower buds but decreased after flower opening, consistent with the relative high presence of (E)-atlantone, kessane, and neointermedeol in flower buds (Figure 2). Conversely, the expression levels of four GERDs (Cluster-15110.54962, Cluster-15110.54963, Cluster-15110.8505, and Cluster-25797.0) were consistent with the high content of 3-buten-2-one,4-(4-hydroxy-2,2,6-trimethyl-7-oxabicyclo[4.1.0]hept-1-yl)- in blooming flowers. For monoterpene biosynthesis, the expression of one geranylgeranyl diphosphate synthase gene (GPPS, Cluster-15110.50692) and three (+)-neomenthol dehydrogenase genes (ND, Cluster-15110.28505, Cluster-15110.47193, and Cluster-15110.47332) increased as the flowers gradually bloomed and maintained high expression levels in blooming flowers, consistent with the contents of 3-isopropenyl-trans-8a-methyl-6-oxo-perhydro-trans-4a-naphthol, 6-octen-1-ol, 7-methyl-3-methylene-, albene, bicyclo[2.2.1]heptane, 7,7-dimethyl-2-methylene-, and citronellol in BF flowers.

**Figure 5:**
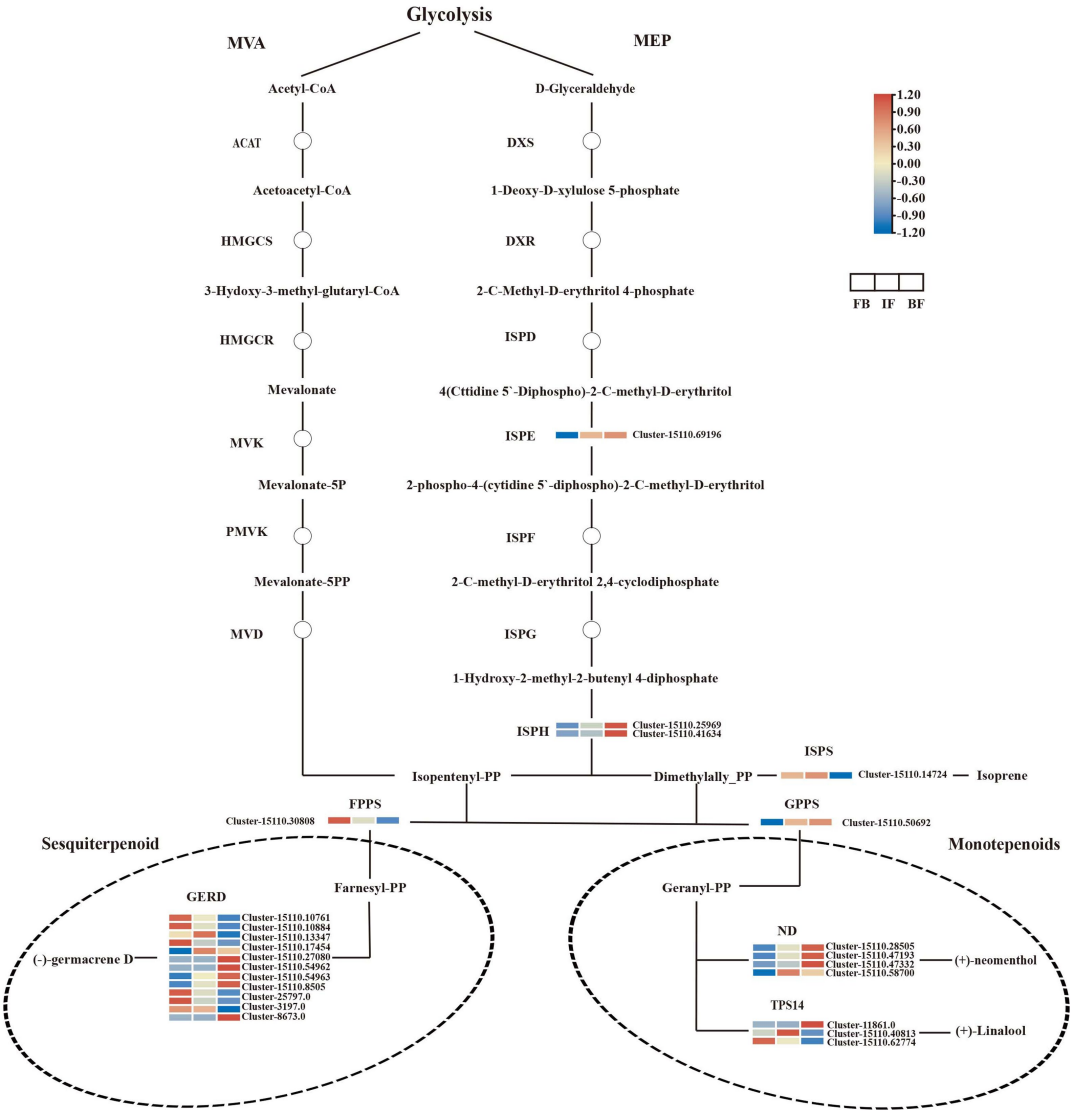
Expression heatmaps of genes associated with terpenoid biosynthesis in *Chrysanthemum indicum* var. *aromaticum* flowers at three developmental stages.

Terpene synthases (TPSs) are key drivers for the generation of terpene scaffold diversity. In the present study, three linalool synthase genes (TPS14, Cluster-11861.0, Cluster-15110.40813, and Cluster-15110.62774) were identified, and each of them was significantly upregulated at three different flowering stages. The expression levels of one TPS14 gene (Cluster-11861.0) were consistent with the accumulation of linalool at the BF stages (Figures 2, 5; Supplementary Tables 2, 15). Comparatively, more DEGs were involved in the biosynthesis of sesquiterpenes rather than monoterpenes, consistent with more sesquiterpenes were indentified during the three CIA flower developmental stages (Supplementary Table 3).

### Genes relating to volatile phenylpropanoids and esters biosynthesis

**Figure 6:**
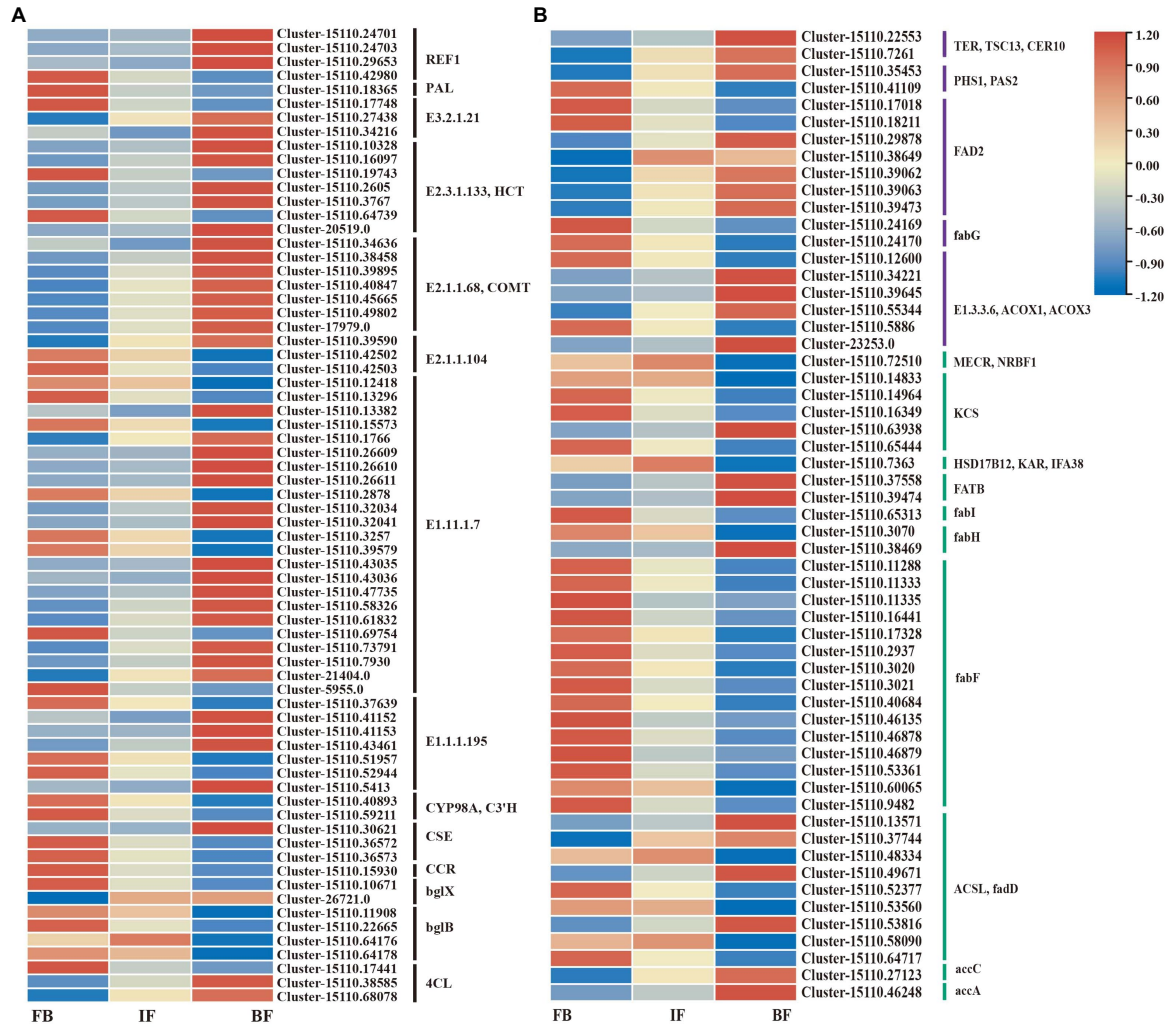
Expression heatmaps of candidate genes involved in volatile metabolite biosynthesis in flowers of *Chrysanthemum indicum* var. *aromaticum*. **(A)** Phenylpropanoid-related pathway. **(B)** Ester metabolic pathway (purple and green).

Based on gene functional annotations, 70 DEGs encoding 14 known enzymes were assigned to the phenylpropanoid biosynthesis pathway (Figure 6; Supplementary Table 16). The expression patterns of these genes were variable across the different flower developmental stages. 11 genes showed significantly higher expression levels in the FB samples but decreased expression in both IF and BF samples, consistent with the changed trend in the contents of some esters such as benzenepropanoic acid and eugenol. By contrast, 16 DEGs showed higher expression levels in the BF samples, consistent with the relatively high content of 2-propenoic acid, 3-phenyl-, methyl ester in this stage (Figures 2, 6A; Supplementary Table 2). In general, genes with significantly high expression levels were mainly concentrated in both the CIA flowers buds and blooming flowers.

Similar gene expression trends were observed for unsaturated fatty acid and fatty acid biosynthesis pathways, in which significantly upregulated genes were less abundant at the IF stage (Figure 6B; Supplementary Tables 12–14). For the unsaturated fatty acid biosynthetic pathway, seven genes in flower buds clearly showed higher expression levels, and 11 genes showed increased expression levels in blooming flowers (Supplementary Table 17). For the fatty acid biosynthesis pathway, 20 genes showed higher expression levels in flower buds, and nine genes showed increased expression in blooming flowers (Supplementary Table 18).

### Transcription factors involved in the biosynthesis of aroma compounds

We calculated the Pearson’s correlation coefficients between the 89 key differential metabolites narrated above and 399 TFs, which were significantly differential expression across the three different flower developmental stages of CIA (Supplementary Table 19). There were 24 TFs whose correlation coefficients were 0.36–0.98 and significantly positively related to 58 differential metabolites (Supplementary Table 20). These TFs belong to the families of MYB (9), bHLH (8), AP2/EFR (5), WRKY (2), and they had significantly high expression levels in FB flowers.

**Figure 7:**
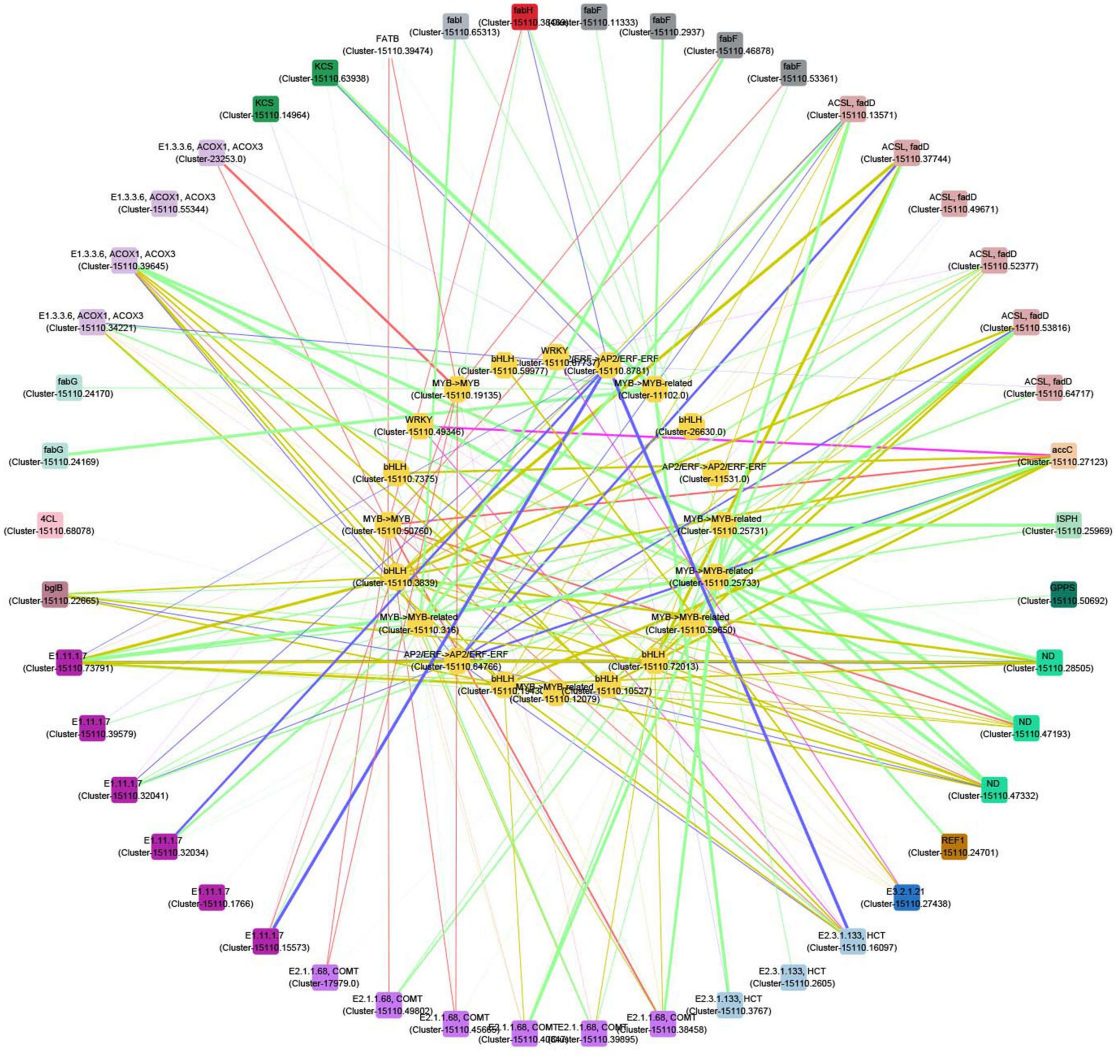
Correlation network between transcription factors (TFs) and structural genes in the biosynthesis of terpenes, phenylpropanoids, unsaturated fatty acids, and fatty acids. Outer circle: structural genes; inner circle: TFs; color of nodes: gene types; width of edges: correlation coefficient (0.9–1).

A correlation network was further plotted to discover the potential regulatory mechanisms between the 24 TFs and the related structural genes which contributed to the biosynthesis of volatile compounds. There were 20 TFs whose correlation coefficients were greater than 0.9 (Figure 7). Most of these TFs are highly related to genes involved into the biosynthesis of terpenes, phenylpropanoids, unsaturated fatty acids, and fatty acids. For example, the expressions of two MYB (Cluster-15110.25731 and Cluster-15110.25733) and one MYB (Cluster-15110.59650) genes are related to those of one ISPH (Cluster-15110.25969) and GPPS (Cluster-15110.50692) genes, respectively. Four MYB (Cluster-15110.25731, Cluster-15110.25733, Cluster-15110.316, and Cluster-15110.50760), four bHLH (Cluster-15110.10527, Cluster-15110.19430, Cluster-15110.3839, and Cluster-15110.72013), and one AP2/EFR (Cluster-15110.64766) genes are highly related to three ND (Cluster-15110.28505, Cluster-15110.47193, and Cluster-15110.47332) genes. These genes are thus the potential regulators contributing to the biosynthesis of terpenes.

### Real time-quantitative PCR validation of representative DEGs

To examine the expression level of selected genes involved in floral scent and flavonoid production, 9 DEGs were chosen from the RNA-seq data for qRT-PCR analysis. As a result, the relative expression trends of these genes examined by qRT-PCR were found to be highly consistent with the RNA-seq analysis (Supplementary Figure 6). FPPS (Cluster-15110.30808), TPS14 (Cluster-15110.62774), and 4CL (Cluster-15110.17441), showed a similarly high levels in flower buds that decreased after flower opening. Conversely, the transcript level of TPS14 (Cluster-11861.0) was similar to those of COMT (Cluster-15110.38458), 4CL (Cluster-15110.38585), FATB (Cluster-15110.39474), and fabH (Cluster-15110.38469), with significantly higher expression during the BF stage. These qRT-PCR results suggested that the gene expression profiles from RNA-Seq data were reliable and reproducible.

## Discussion

### Main volatile compounds of CIA fresh flowers

The intense, whole-plant fragrance of CIA is a key characteristic that distinguishes it from other chrysanthemums, including its original variety, *C. indicum*. In this study, a total of 337 volatile secondary metabolites were identified from fresh flower samples of CIA at three different developmental stages (Supplementary Table 2). The majority of these volatiles were terpenes and esters, similar to those previously investigated in essential oils of CIA (Lin et al., 2016). However, the total number of volatile compounds identified in the present study is much more than those in previous studies. For example, using the same GC–MS method, an analysis of volatile compounds in the essential oils of CIA identified 44 volatile metabolites, which accounted for over 43% of the total volatile oil (Lu and Li, 2002). The main constituents in the essential oils were monoterpenes, sesquiterpenes, and oxygen-containing derivatives, such as verbenol, (−)zingiberene, β-sesquiphellandrene, farnesene, trans-chrysanthenyl acetate, and caryophyllene (Lu and Li, 2002). Further analysis of hydro-distillation oil from dried flowers of CIA identified 50 volatile compounds, including sabinol (14.3%), carveol (8.9%), and β-patchoulene (5.9%; He et al., 2000). Similarly, a parallel study identified 52 volatile organic compounds in CIA buds and flowers (Xie et al., 2011).

The main aroma substances of fresh CIA samples also differ from those of essential oils and dried flowers. Lin et al. established a GC–MS fingerprinting method for volatile oils extracted from CIA flowers and detected 23 main characteristic components (Lin et al., 2016). The content of acetic acid oxidized linalool ester was as high as 20%, and it was the most significant index compound in the volatile oils of CIA flowers (Lin et al., 2016). Another study showed that the chemical constituents of CIA dried flower oils consisted mainly of fatty acid derivatives, monoterpenes, sesquiterpenes, and oxygen-containing compounds, and the content of oxygen-containing compounds was much larger than that of non-oxygen-containing compounds (Wang et al., 1986). By comparison, the main aroma components in the fresh CIA samples investigated here were benzene,1-methyl-3-(1-methylethyl)-, azacyclotridecan-2-one, linalool, caryophyllene, α-thujone, and β-thujone (Supplementary Table 19).

### Changes in floral scent during different flower developmental stages

The release of floral fragrance can continuously change over the course of flower opening (Li et al., 2006). For example, a study of the aroma component emission pattern of Osmanthus fragrans ‘Boye Jingui’ showed that the most intense release of floral fragrance occurred at the initial bloom stage (Shi et al., 2018). In the present study, most of the volatiles were released at the bud stage of CIA flowers, and the composition and release of volatiles changed as the flowers bloomed. As the flowers gradually opening, the release of most volatile substances, including lipids, aldehydes, alcohols, ketones, and terpenes, showed a decreasing trend. This result was similar to an analysis of aroma compounds and their release from different flowering stages of the Chrysanthemum cultivar ‘Jinba’ (Jin et al., 2012).

The characteristic floral fragrance components of CIA also changed across flower developmental stages. The skeleton types of esters in CIA flowers are abundant and diverse; the main types at the BF stage are alkane skeleton esters, whereas benzene ring skeleton esters are present mainly at the IF stage (Figure 2B). Among the terpenes, monoterpenes are predominant at the BF stage, whereas sesquiterpenes are predominant at the IF stage. Some volatiles appear only in the flower buds of CIA; some others are synthesized from the flower buds, and most of them are released during the flowering stages. Watanabe and Moon studied the dynamic changes in glycoside precursors in the aroma of jasmine flowers; they reported that the release of benzyl alcohol, linalool, methyl anthranilate, and other substances occurred 1–12 h after flowering, then decreased slightly to 24 h after flowering (Moon et al., 1994; Watanabe et al., 2014). The decline in aroma release may be related to the rapid decrease in enzyme activity during flower wilting.

Floral volatiles are significant in biotic interactions, especially for pollinators attractions and defense functions (Muhlemann et al., 2014). Of which, benzenoids mostly serve in pollinator-attracting whereas terpenoids and benzenoids predominantly mediate prevention of damage to their reproductive structures (Schiestl, 2010; Nagegowda and Gupta, 2020). To maximize reproductive successs, plant flowers generally employed several strategies of adaptive mechanisms to balance attracting and preventing functions of floral volatiles, lean heavily on amount, composition, context, and timing of their emission (Theis and Adler, 2012; Muhlemann et al., 2014). For instance, within the petunia floral volatiles, methylbenzoate have been described as involving in pollinator-attracting whereas some compounds particularly mediate infestation from florivores (i.e., isoeugenol and benzylbenzoate; Andrews et al., 2007; Kessler et al., 2013). In this study, the release of five monoterpenes and three sesquiterpenes showed a decreasing trend during flower blooming, and 4-isopropyl-1-methylcyclohex-2-enol, (1S,1aS,1bR,4S,5S,5aS,6aR)-1a,1b,4,5a-tetramethyldecahydro-1,5-methanocyclopropa[a]indene, and benzaldehyde, 4-(1-methylethyl)- were not even detected at the FB stage. We found that the concentration of 17 aromatic substances increased significantly with flower blooming. Although the concentrations were generally low, they were highly recognizable, enabling CIA flowers to fully prepare for attraction of potential insect pollinators and also to resist pathogens.

### Expression pattern of floral scent-related genes in CIA

The formation and release mechanism of floral fragrance is complex and depends on the biosynthetic pathway of aroma compounds and the activities of related enzymes (Pichersky and Dudareva, 2007; Maeda and Dudareva, 2012). In this study, 104,165 expressed genes were identified from CIA flower–derived transcriptome data, and 8,945 DEGs were identified between samples from different flower developmental stages (Figure 4B). Pairwise comparisons showed that the number of upregulated DEGs was higher than that of downregulated DEGs (FB *vs*. IF: 568 up/79 down; IF *vs*. BF: 1423 up /1282 down; FB *vs*. BF: 5006 up /3591 down), suggesting that the diversity and production of fragrance compounds during different flower developmental stages are related to the activation/ inhibition of these DEGs.

For the terpene biosynthesis pathway, no DEGs were detected in the MVA upstream pathway, whereas two and 16 DEGs were identified in the DXP/MEP upstream and downstream pathways, respectively. The expression levels of five GERD genes (Cluster-15110.10761, Cluster-15110.10884, Cluster-15110.17454, Cluster-3197.0, and Cluster-8673.0) were consistent with the present levels of (E)-atlantone, kessane, and neointermedeol, which were abundant in flower buds but decreased following flower opening. Conversely, the expression patterns of four other GERD genes (Cluster-15110.54962, Cluster-15110.54963, Cluster-15110.8505, and Cluster-25797.0) were consistent with the levels of 3-buten-2-one and 4-(4-hydroxy-2,2,6-trimethyl-7-oxabicyclo[4.1.0]hept-1-yl)-, which were detected mainly in blooming flowers. This result suggests that different GERD homologues may have divergent functions and expression levels, leading to the production of different compounds. A similar phenomenon was observed in grapevine (Vitis vinifera): VvGERD genes had lower transcript levels in open, pre-anthesis flowers, flowers after anthesis, or at the early onset of fruit development, highlighting the regulatory role of VvGERDs in sesquiterpene production during flower and fruit development in grapevine (Lücker et al., 2004). The activity of GPPS also affects the synthesis of other products in the isoprene pathway (Kumari et al., 2013). Our transcription data revealed one GPPS gene (Cluster-15110.50692) and three ND genes (Cluster-15110.28505, Cluster-15110.47193, and Cluster-15110.47332) that were similar to ISPE and ISPH; they reached maximum expression levels at the BF stage, consistent with the contents of 3-isopropenyl-trans-8a-methyl-6-oxo-perhydro-trans-4a-naphthol, 6-octen-1-ol, 7-methyl-3-methylene-, albene, bicyclo[2.2.1]heptane, 7,7-dimethyl-2-methylene-, and citronellol. In Hedychium coronarium, HcGPPS also showed an expression trend similar to that of some monoterpenes, demonstrating an important regulatory role for GPPS in monoterpene biosynthesis during flower development (Yue et al., 2015). In addition, endo-borneol showed the highest emission rate in the borneol chemotype of Cinnamomum camphora owing to the high expression of ND, which displayed seasonal regulation (Tian et al., 2021). Therefore, GPPS and ND may also play an important developmental regulatory role in the synthesis of monoterpenes in CIA.

Many terpenes in floral compounds are direct products of TPS, while others are formed by hydroxylation, dehydrogenation, acylation, and other reactions based on the formation of terpenoids catalyzed by TPSs (Aharoni et al., 2006). In this study, the transcript abundance of one TPS14 (Cluster-15110.62774) decreased as flowers gradually bloomed, yet the expression of another TPS14 (Cluster-11861.0) showed the opposite pattern, with higher expression levels in blooming flowers, consistent with the accumulation of linalool in different flowering stages (Figures 2B, 5; Supplementary Table 1). Among the four chemotypes of C. camphora, the linalool chemotype showed the highest emission rate of monoterpenes in the same month, owing to the high expression of genes in the MEP pathway, an α-terpineol synthase gene (E4.2.3.111) and TSP14, which were possibly responsible for the generation of volatile monoterpenes (Tian et al., 2021). Therefore, TPS plays an important role in the biosynthesis of volatile terpenes in CIA flowers.

Polyphenols, an abundant population of secondary metabolites in plants, are derived from the phenylpropanoid pathway (Dudareva and Pichersky, 2000). This pathway is a rich source of plant metabolites and the starting point for the production of a variety of important plant compounds, including flavonoids, coumarin, and lignans (Negre, 2005). The profile and concentration of phenylpropanoids vary in different tissues, different stages of growth and development, and even in different eco-geographical groups of the same species. Several studies have found that PAL, C3H, C4H, and 4CL are the key enzymes for the biosynthesis and accumulation of phenylpropanoids (Boatright et al., 2004; Zhao et al., 2017). In the present study of flower buds, the expression of 11 DEGs involved in the biosynthesis of phenylpropanoids and flavonoids, including one PAL, two CYP98A/C3Hs, and one 4CL, decreased as the flowers gradually bloomed. By contrast, increased expression levels of 15 DEGs were observed in blooming flowers, including two 4CL. These observations suppose that the diverse expression of these DEGs in branches of the phenylpropanoid pathway may mainly leading to the production of scent in the CIA during flower development (Zvi et al., 2012). Fatty acid derivatives are mainly formed through three processes: α oxidation, β oxidation, and the lipoxygenase pathway. C18 fatty acids (linoleic acid and linoleic acid) are transformed into hydroperoxidates in the presence of lipoxygenase (LOX; Schwab et al., 2008). The high content of 28 esters in flower buds may be related to the upregulated expression of 26 DEGs involved into the biosynthesis of saturated and unsaturated fatty acids, and the relatively high content of seven esters in blooming flowers may be related to the upregulated expression of 20 DEGs.

In this study, we found 20 TFs were positively related to the expressions of genes involved into the biosynthesis of terpenes, phenylpropanoids, unsaturated fatty acids, and fatty acids (Figure 7). Morever, many of these TFs were also highly related to the presence of the relative contents of these compounds in different CIA flower developmental stages (Supplementary Table 19). In rose flowers, production of anthocyanin pigment1 (PAP1) was confirmed to regulate the synthesis of terpenoids and benzenoid compounds (Zvi et al., 2012). These TFs herein may interact with promoters of synthase genes of related compounds or regulate upstream compounds to affect metabolic flow. These results further suggest that the activity of these DEGs and TFs may contribute to the diversity and release of floral substances at different flowering stages of CIA. The specific sources of characteristic aroma compounds and regulatory mechanism require further study.

## Conclusion

We performed integrative metabolome and transcriptome analyses to investigate the molecular mechanisms underlying the production of volatile metabolites by CIA flowers at three different developmental stages. The presented metabolic profile showed clearly developmental stage-specific patterns, in which both volatile terpenes and esters are highly enriched and varied across different flower developmental stages.Moreover, a number of DEGs revealed a similar pattern, in which the expressions of different genes were significantly upregulated or downregulated from FB to BF stages, and many of them came from the pathways responsible for the biosynthesis of terpenoids, esters, and phenylpropanoids. TFs, including those from MYB, bHLH, AP2/EFR and WRKY families, were also found to be involved into the regulation for generating CIA floral scent. The results presented in this study will facilitate floral scent-related gene discoveries and help to enable the molecular breeding and metabolic engineering of chrysanthemum plants. Moreover, because CIA is a valuable resource plant, these results will further promote its agricultural, medicinal, and industrial applications in the future.

## Data availability statement

The data presented in the study are deposited in the SPA repository, accession number PRJNA792202.

## Author contributions

BH and JZ: conceptualization. LZ, JL, CZ, and XW: investigation. JL, LZ, and JZ: formal analysis. ZH and BH: resources. LZ, JZ, and BH: writing‑original draft preparation. JZ, BH, and YL: writing‑review and editing. All authors have read and agreed to the published version of the manuscript.

## Funding

This work was supported by Natural Science Foundation of China, Grant/Award Numbers: 31900267; National Key Research and Development Program of China, Grant/Award Numbers: 2019YFC1711100; Central Government Guides Local Science and Technology Development Fund in Hubei Province, Grant/Award Numbers: 2019ZYYD063; Hubei Science Foundation for Distinguished Young Scholars, Grant/Award Numbers: 2019CFA097.

## Conflict of interest

The authors declare that the research was conducted in the absence of any commercial or financial relationships that could be construed as a potential conflict of interest.

## Publisher’s note

All claims expressed in this article are solely those of the authors and do not necessarily represent those of their affiliated organizations, or those of the publisher, the editors and the reviewers. Any product that may be evaluated in this article, or claim that may be made by its manufacturer, is not guaranteed or endorsed by the publisher.

## Footnotes

^1^ https://github.com/CJ-Chen/TBtools/releases

^2^ http://trinityrnaseq.github.io

^3^ https://code.google.com/p/corset-project/

^4^ http://bioinformatics.psb.ugent.be/webtools/Venn/

## Supplementary material

SUPPLEMENTARY FIGURE 1 CIA flowers from different developmental stages: flower bud (FB), initial flower (IF), and blooming flower (BF).

SUPPLEMENTARY FIGURE 2 Expression heatmaps of genes detected in flowers of *Chrysanthemum indicum* var. *aromaticum*.

SUPPLEMENTARY FIGURE 3 KEGG enrichment analyses of DEGs identified in the FB *vs*. IF dataset.

SUPPLEMENTARY FIGURE 4 KEGG enrichment analyses of DEGs identified in the FB *vs*. BF dataset.

SUPPLEMENTARY FIGURE 5 KEGG enrichment analyses of DEGs identified in the IF *vs*. BF dataset.

SUPPLEMENTARY FIGURE 6 qRT-PCR analysis of 12 DEGs chosen from the *Chrysanthemum indicum* var. *aromaticum* RNA-seq data.
