# Supplementary figures and images for: Integrative metabolome and transcriptome analyses reveal the molecular mechanism underlying variation in floral scent during flower development of Chrysanthemum indicum var. aromaticum
Source: Front Plant Sci. 2022 Sep 15;13:919151. doi: 10.3389/fpls.2022.919151 (PMC9889088; doi:10.3389/fpls.2022.919151)

1 cm

**FB**

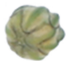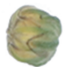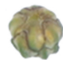

**IF**

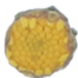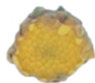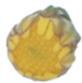

**BF**

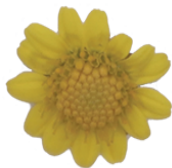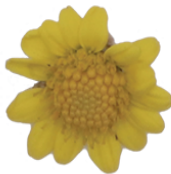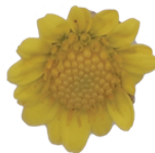

Supplement: SUPPLEMENTARY FIGURE 1 — CIA flowers from different developmental stages: flower bud (FB), initial flower (IF), and blooming flower (BF). [file Data_Sheet_1.PDF]

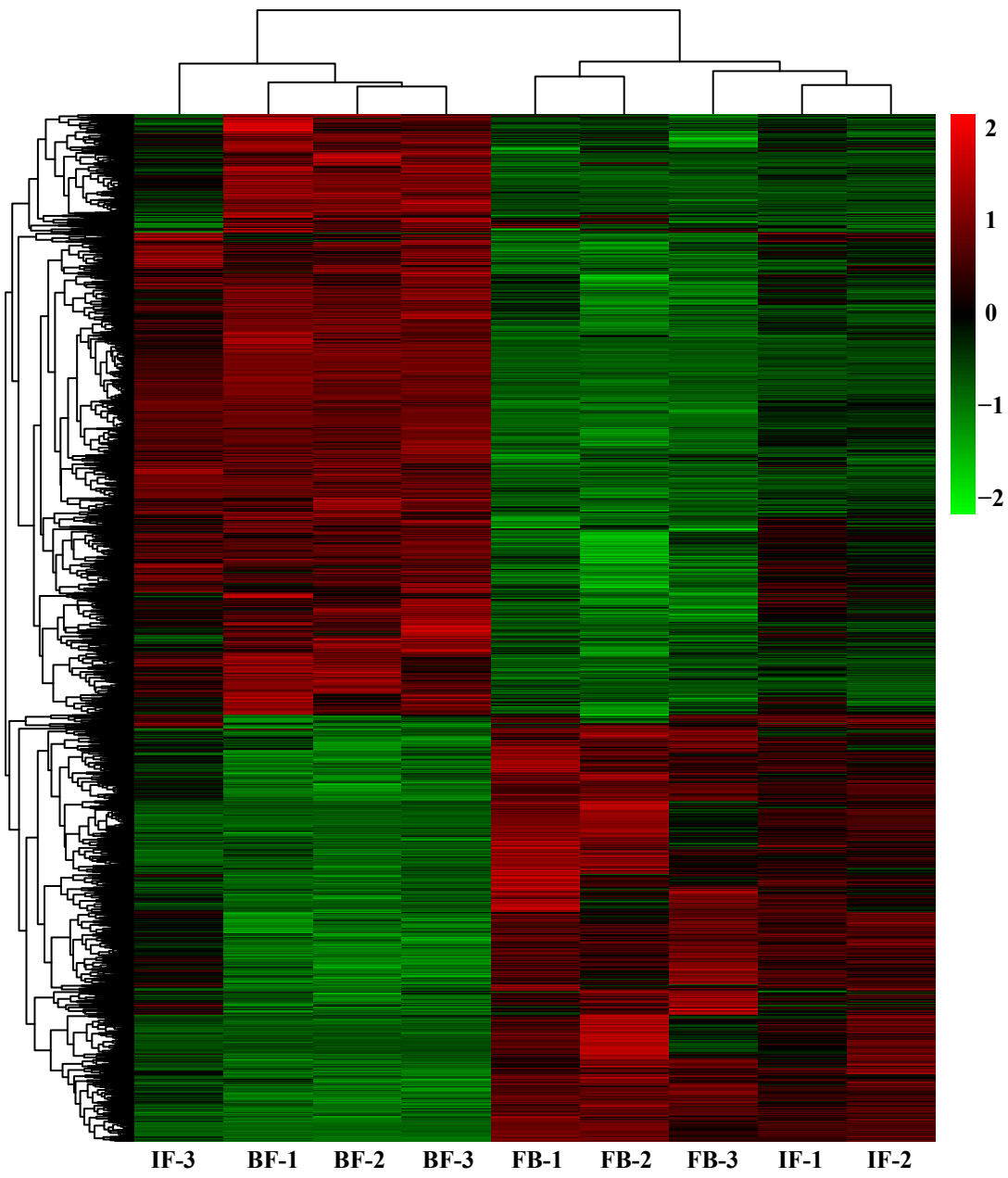

Supplement: SUPPLEMENTARY FIGURE 2 — Expression heatmaps of genes detected in flowers of Chrysanthemum indicum var. aromaticum. [file Data_Sheet_2.PDF]

# Statistics of Pathway Enrichment

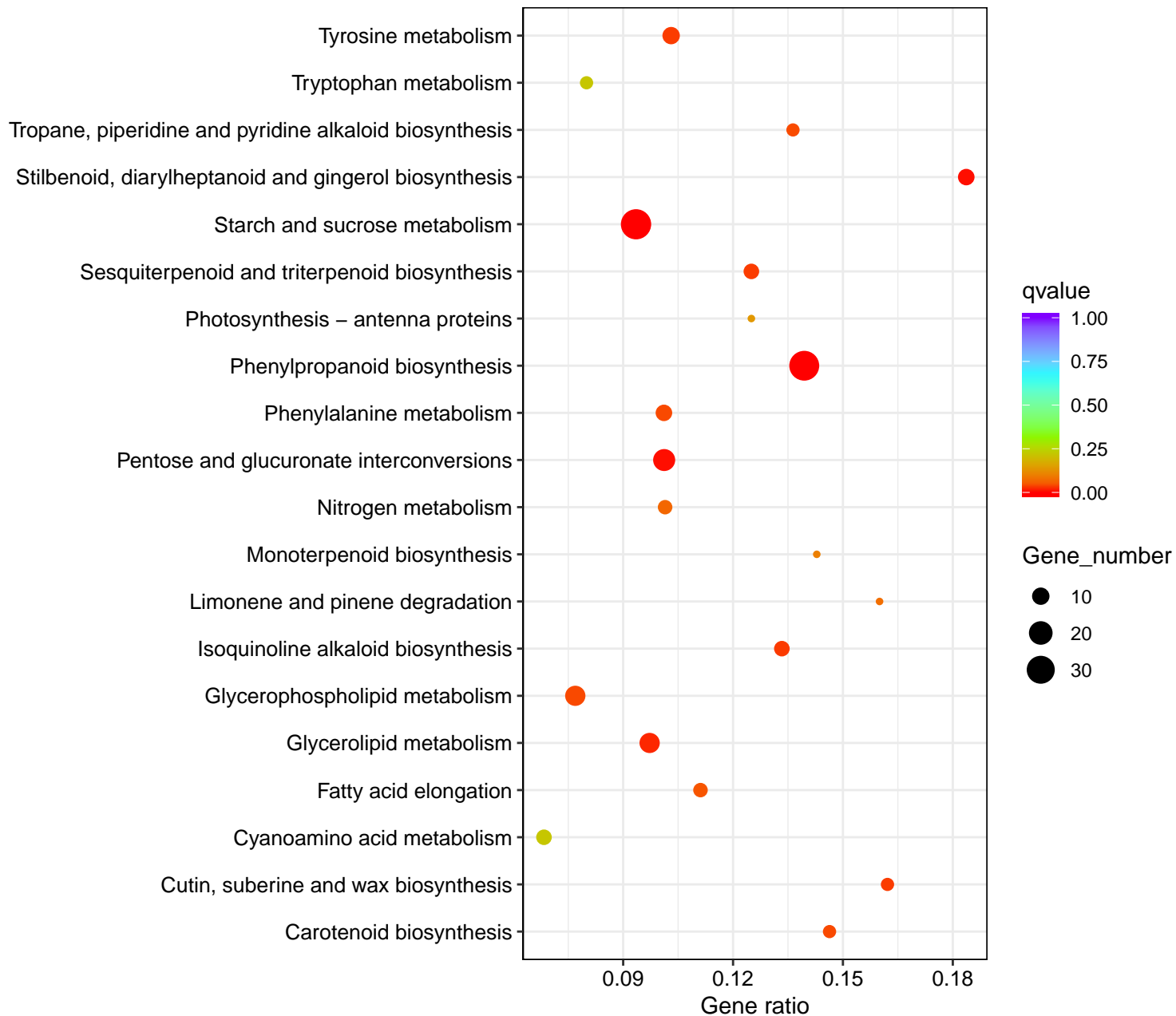

Supplement: SUPPLEMENTARY FIGURE 3 — KEGG enrichment analyses of DEGs identified in the FB vs. IF dataset. [file Data_Sheet_3.PDF]

# Statistics of Pathway Enrichment

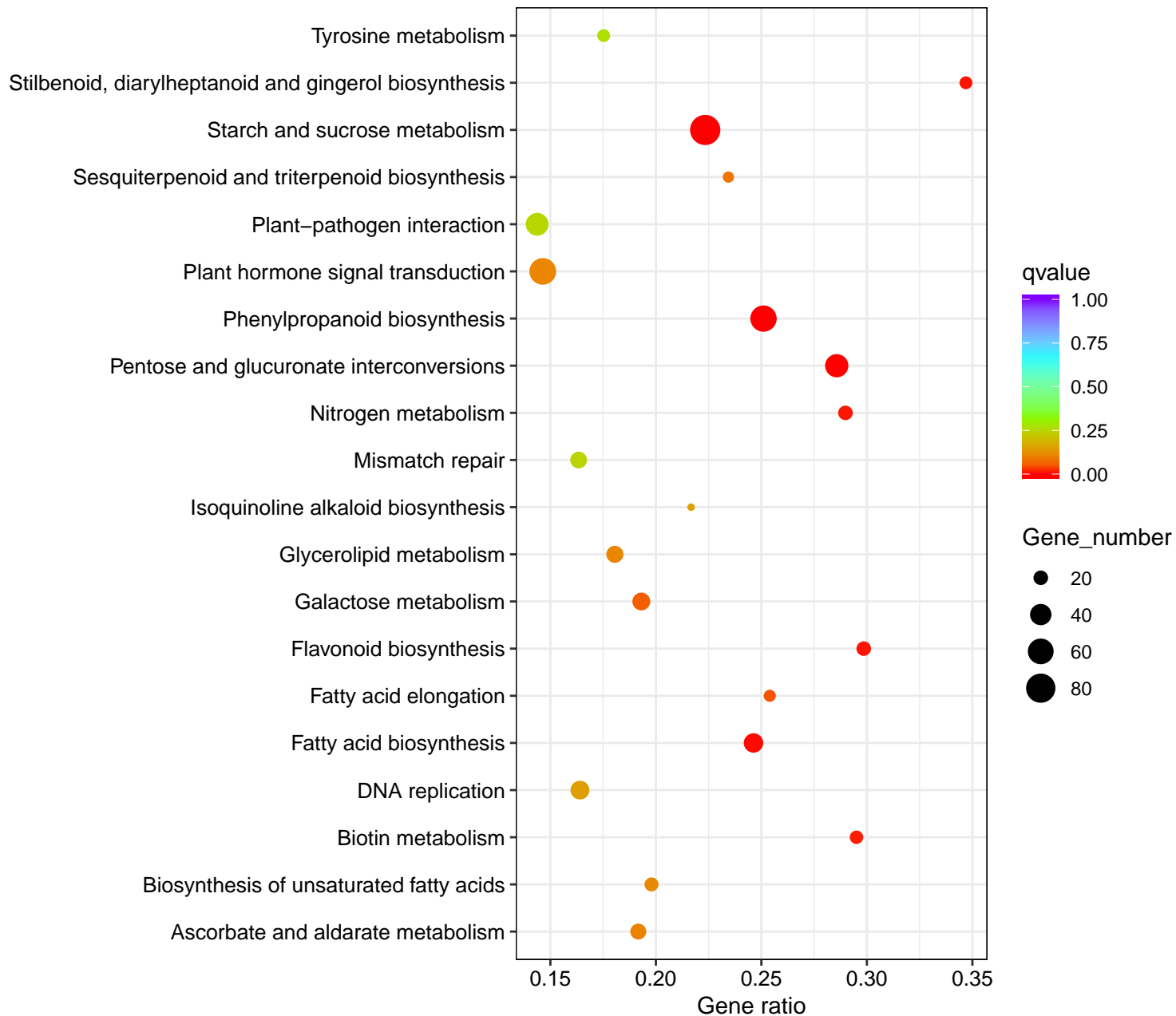

Supplement: SUPPLEMENTARY FIGURE 4 — KEGG enrichment analyses of DEGs identified in the FB vs. BF dataset. [file Data_Sheet_4.PDF]

# Statistics of Pathway Enrichment

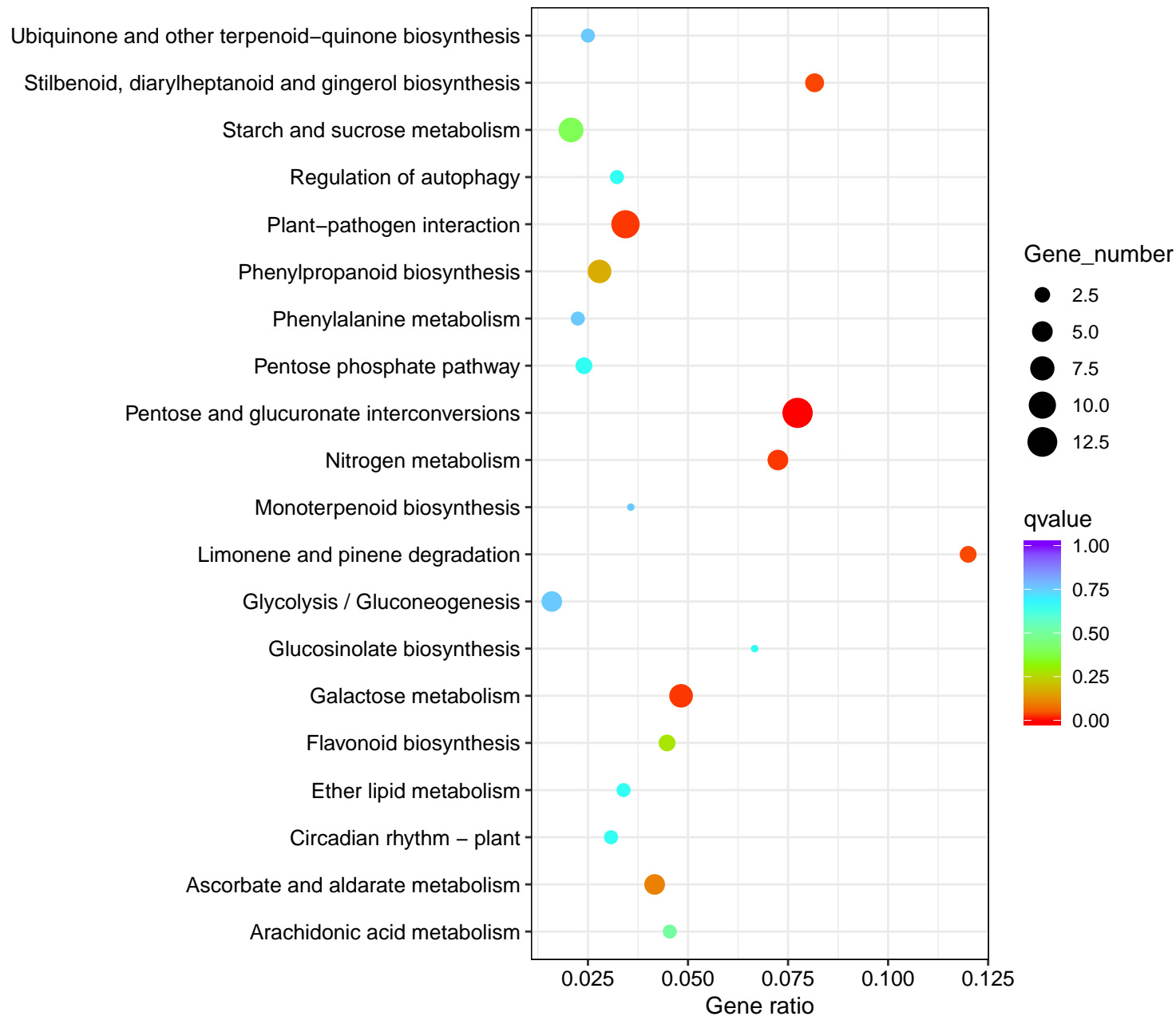

Supplement: SUPPLEMENTARY FIGURE 5 — KEGG enrichment analyses of DEGs identified in the IF vs. BF dataset. [file Data_Sheet_5.PDF]
