# Supplementary material for: Integrative metabolome and transcriptome analyses reveal the molecular mechanism underlying variation in floral scent during flower development of Chrysanthemum indicum var. aromaticum
Source: Front Plant Sci. 2022 Sep 15;13:919151. doi: 10.3389/fpls.2022.919151 (PMC9889088; doi:10.3389/fpls.2022.919151)
Supplement: SUPPLEMENTARY FIGURE 6 — qRT-PCR analysis of 12 DEGs chosen from the Chrysanthemum indicum var. aromaticum RNA-seq data. [file Data_Sheet_6.PDF]

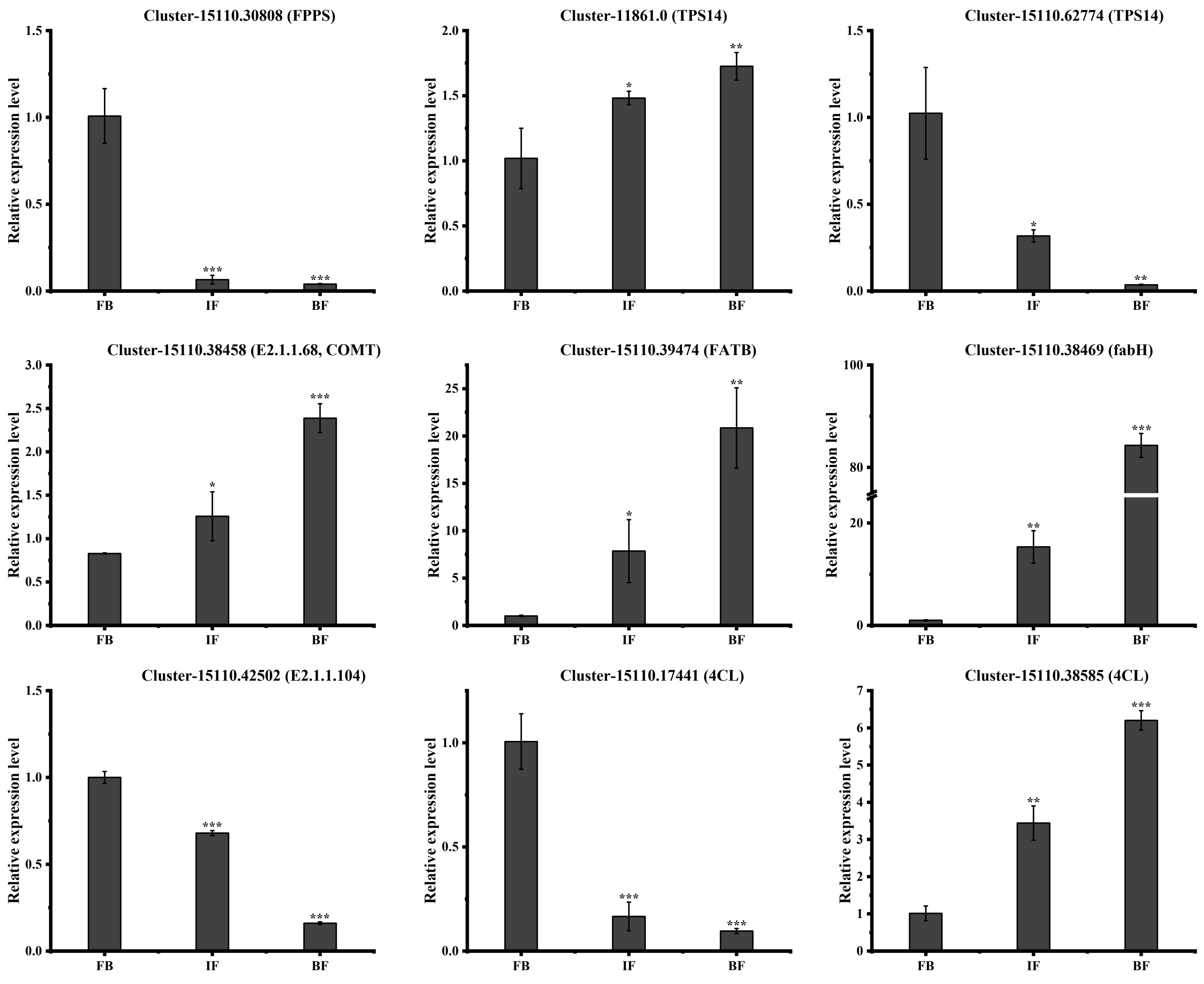

Cluster-15110.30808 (FPPS)
Cluster-11861.0 (TPS14)
Cluster-15110.62774 (TPS14)
Cluster-15110.38458 (E2.1.1.68, COMT)
Cluster-15110.39474 (FATB)
Cluster-15110.38469 (fabH)
Cluster-15110.42502 (E2.1.1.104)
Cluster-15110.17441 (4CL)
Cluster-15110.38585 (4CL)
Relative expression level
FB
IF
BF
